# Cough and Vomiting of the Tuberculous

**Published:** 1913-12

**Authors:** 


### ABSTRACTS

COUGH AND VOMITING OF THE TUBERCULOUS. Dr. H. Paillard. After prolonged and careful investigations on this question, Dr. H. Paillard has come to conclusions which are greatly different from the classical views. About etiology, several factors have to be considered: 1st, the overloading of the stomach; big meals bring on Morton's cough, therefore the patient must be directed to adopt fragmentary feeding; 2d, the fatigue after the meals. This cough is often met with among laborers, workmen, etc., but is not frequent among well-to-do people, who can take a complete rest after the meals. 3d, the period of the disease. Morton's cough is much more common in the first stages of phtisis. 4th, the localization of the pulmonary lesions. It is very common in the common phtisis of the apices, but very rare when pleurisy has already prevented the movements of the left side of the diaphragm. 5th, the "status dyspepticus." This was considered by all the classical writers as an essential factor, but the author thinks it is a mere accessory because it is often absent, and, furthermore, cough is not at all constant in all dyspeptic patients.

There are three varieties of Morton's cough. The most typical is the variety which follows on the onset of phtisis; the cough of the later stages of the disease, less frequent, less regular, and more painful. Lastly the variety which bears no relation to meals but occurs in the morning, especially in patients with pharyngitis, and is not at all special to consumptive patients. The author insists on the feeling of breathlessness which appears after the meals and before the cough.

The pathogeny of Morton's cough has been very much discussed, and the author discusses it again. He is in favor of the mechanical origin of this cough and shows how Peter's theory is vague and feeble. According to H. Paillard the vomiting is due, as in whooping cough, to the jolts and jerks of the cough, and in this respect he insists on the importance of the condition of the diaphragm in the causation of Morton's cough; experimentally he has noticed that the fixation of the left side of the diaphragm almost prevents vomiting, clinically he has seen that Morton's cough seldom occurs in patients whose left side of the diaphragm has been fixed by a former pleurisy. Conversely consumptive patients whose left diaphragm has normal or exaggerated movements when breathing or coughing are very often afflicted by the stiffness of the diaphragm and the "thoracic aspiration" (Arnozan) is deficient; in the second case, i.e., exaggerated movements, the stomach is directly injured and the "thoracic aspiration" is maximum.

As to treatment H. Paillard recommends to give after each meal a few whiffs of oxygen; these inhalations may be repeated if necessary; they relieve the dyspnœa which is so common after the meals and reduces the desire to increase the expansion of the thorax and diaphragm; thus the stomach is given sufficient rest to evacuate its contents at a normal rate. This method, as well as the recommendations which we have seen about etiology, has given excellent results.

